# Hyperspectral Inversion of Soil Organic Matter Content Based on a Combined Spectral Index Model

**DOI:** 10.3390/s20102777

**Published:** 2020-05-13

**Authors:** Lifei Wei, Ziran Yuan, Zhengxiang Wang, Liya Zhao, Yangxi Zhang, Xianyou Lu, Liqin Cao

**Affiliations:** 1 Faculty of Resources and Environmental Science, Hubei University, Wuhan 430062, China; weilifei2508@hubu.edu.cn (L.W.); wangzx66@hubu.edu.cn (Z.W.); zhaoly0128@hubu.edu.cn (L.Z.); 201811110811176@stu.hubu.edu.cn (Y.Z.); 201911110811278@stu.hubu.edu.cn (X.L.); 2 Hubei Key Laboratory of Regional Development and Environmental Response, Hubei University, Wuhan 430062, China; 3 Key Laboratory of Urban Land Resources Monitoring and Simulation, Ministry of Land and Resources, Shenzhen 518034, China; 4 Institute of Soil and Fertilizer, Anhui Academy of Agricultural Sciences, Hefei 230031, China; 5 School of Printing and Packaging, Wuhan University, Wuhan 430079, China; clq@whu.edu.cn

**Keywords:** hyperspectral remote sensing, soil organic matter, AdaBoost algorithm, pearson correlation analysis

## Abstract

Soil organic matter (SOM) refers to all carbon-containing organic matter in soil and is one of the most important indicators of soil fertility. The hyperspectral inversion analysis of SOM traditionally relies on laboratory chemical testing methods, which have the disadvantages of being inefficient and time-consuming. In this study, 69 soil samples were collected from the Honghu farmland area and a mining area in northwest China. After pretreatment, 10 spectral indicators were obtained. Ridge regression, kernel ridge regression, Bayesian ridge regression, and AdaBoost algorithms were then used to construct the SOM hyperspectral inversion model based on the characteristic bands, and the accuracy of the models was compared. The results showed that the AdaBoost algorithm based on a grid search had the best accuracy in the different regions. For the mining area in northwest China, $R_{p}^{2}$ = 0.91, $RMSE_{p}$ = 0.22, and $MAE_{p}$ = 0.2. For the Honghu farmland area, $R_{p}^{2}$ = 0.86, $RMSE_{p}$ = 0.72, and $MAE_{p}$ = 0.56. The detection of SOM content using hyperspectral technology has the characteristics of a high detection precision and high speed, which will be of great significance for the rapid development of precision agriculture.

## 1. Introduction

Soil organic matter (SOM) is an important part of soil. It promotes plant growth and improves the physical properties of soil. It plays a very important role in promoting the formation of soil structure, improving the physical properties of soil and soil’s ability to retain fertilizer, and is the main limiting factor for crop yield [1,2]. Therefore, rapid and accurate monitoring of SOM is of great significance for soil fertility monitoring and agricultural development. The traditional methods for the determination of soil organic matter are still widely used because of their high precision. However, these methods are usually time-consuming, laborious, harmful, or polluting, and it is difficult to directly determine results in the field. Additionally, the traditional method is based on point measurement, which has few measuring points, a slow speed, a limited scope, and cannot meet the requirements of precision fertilization technology and precision agriculture regarding the spatial-temporal variation of soil organic matter [3]. The methods based on spectroscopy have the characteristics of high efficiency, wide coverage, low cost, and they can be used to effectively determine the SOM content. However, in the original spectrum acquisition process, due to factors such as the collection environment and the instrument itself, a large amount of irrelevant redundant information will appear. This greatly affects the prediction accuracy and the robustness of the model. Therefore, data reduction and reduction of unrelated information for the original spectral information is an important prerequisite and the basis for constructing quantitative inversion models [4,5].

Since the 1960s, the quantitative inversion of the reflectance spectra of soil organic matter has been carried out around the world. Nawar et al. [6] studied the soil of the Sinai Peninsula in Egypt, using Savitzky–Golay (SG) smoothing, first-order derivative (FD), second-order derivative (SD), continuum removal (CR), and standard normal variate detrend (SNV-D) transformations. Multiplicative scatter correction (MSC) and extended MSC were used to preprocess the original spectra. Partial least squares regression (PLSR), support vector regression (SVM), and multiple adaptive regression splines (MARS) were used to determine the best method for assessing the SOM and clay content in soil affected by salt. The results showed that the MARS model outperformed the PLSR and SVM models, and the MARS model after a CR treatment obtained the best prediction results. Steinberg et al. [7] analyzed the absorption characteristics of the spectral curves of soils with different organic matter contents and concluded that the PLSR model established by the logarithmic processing of the removal of the envelope line and the reciprocal of the reflectance had the best prediction effect. 

However, in visible and near-infrared spectroscopy (Vis/NIRS, 350–2500 nm), the reflectance of the soil spectrum is generally low. The absorption characteristics are not significant and they are easily affected by the external environment; therefore, the extension of the inversion model established by directly using the measured spectrum is limited. The spectral reflectance of soil is a comprehensive response to the spectral behavior of soil’s inherent physical and chemical properties. Different types of soil have different spectral characteristics due to different physical and chemical properties. Additionally, even for the same type of soil, different parent materials affect their spectral characteristics. Therefore, by studying different types of soil, the prediction model of organic matter content obtained by using the combined spectral index model is of great significance for studying the generalization ability of the model.

Therefore, in this study, we selected the characteristic bands based on different spectral index transformations and the Pearson correlation analysis method, where the bands with a correlation coefficient of greater than 0.7 were selected as the characteristic bands. Then, ridge regression (RR), kernel ridge regression (KRR), bayesian ridge regression (BRR), and adaBoost algorithms were used to establish the inversion model. The quantitative inversion method for SOM lays a foundation for the use of hyperspectral remote sensing imagery for soil fertility monitoring and agricultural development.

## 2. Materials and Methods

### 2.1. Study Areas

Two sites were selected for soil sampling, where one was a mining area and the other was farmland. The mining area soil was collected from northwest China. The area belongs to the northern temperate continental climate. It has rich mineral resources, four distinct seasons, and abundant sunshine. The area has the natural advantages of water, soil, light, and heat resources for the development of agriculture. The main soil types in this area are alluvial soils and irrigated soils, which are formed under the influence of long-term cultivation, fertilization, and irrigation. 

The farmland soil was collected from Yanwo Town of the city of Honghu, China, which is under the jurisdiction of the city of Jingzhou, Hubei province. The economy in this area is mainly based on agriculture, particularly rice, sesame, cotton, wheat, and other crops. The area features a subtropical humid monsoon climate, abundant sunshine, abundant rainfall, and excellent water resources, but floods are prone to occur in summer. Agricultural cultivation in this area has a long history.

### 2.2. Research Methods

#### 2.2.1. Ridge Regression

The ridge regression (RR) method is a biased estimation method for the analysis of multicollinear data [8,9]. At the cost of the partial precision of the least-squares regression equation, a regression equation with a strong tolerance to ill-conditioned data is obtained, which can better solve the problem of the collinearity of hyperspectral data.

(1) $$\beta (k)={\left(X^{T}X+kI_{p}\right)}^{-1}X^{T}Y$$

Here, k is the parameter of ridge estimate, $I_{p}$ is the P-order unit matrix, and p is the number of modeling samples.

#### 2.2.2. Kernel Ridge Regression

Kernel ridge regression (KRR) is a regression analysis model that combines ridge regression with kernel techniques [10]. For a nonlinear kernel, this corresponds to a nonlinear function in the original space. Kernel regression has an approximate solution and is highly efficient when used on moderate-scale data.

(2) $$J=\frac{1}{2}{\sum i{\left(y_{i}-\omega^{T}x_{i}\right)}^{2}+\frac{1}{2}\lambda \left\|\omega \right\|}^{2}$$

Here, λ is the regularization term. Once again, to generalize this model to non-linear cases, a kernel trick is applied, which maps the data into a higher-dimensional space.

#### 2.2.3. Bayesian Ridge Regression

Bayesian ridge regression (BRR) is a machine learning regression algorithm based on Bayesian theory [11,12]. Bayesian linear regression is shown in Equation (3). Its purpose is to find the parameter vector distribution that makes the loss function (Equation (4)) the smallest.

(3) $$y(x,\omega )=\sum_{j=0}^{n}\omega_{j}\psi_{j}(x)=\omega^{T}\psi (x)$$

(4) $$J(\omega )={\sum_{i=1}^{m}\left\{y(x_{i},\omega )-t_{i}\right\}}^{2}$$

Here, *n* is the dimension of sample space, *m* is the sample capacity, ω is the parameter vector, ψ(x) is the nonlinear function of input vector *x*, and the prior probability of ω is given in Equation (5):

(5) $$p(t|\omega )=\frac{1}{2\pi \sigma_{1}^{2}}\exp\left(-\frac{1}{2\sigma_{1}^{2}}\sum_{i=1}^{m}{\left\{y(x_{i},\omega )-t_{i}\right\}}^{2}\right)$$

According to Bayesian rules:

(6) $$p(\omega |t)=\frac{p(\omega )p(t|\omega )}{p(t)}$$

where:

(7) $$p(\omega )=\frac{1}{2\pi \sigma_{2}^{2}}\exp\left(-\frac{1}{2\sigma_{2}^{2}}\omega^{T}\omega \right)$$

such that:

(8) $$\ln(p(\omega |t))=-\frac{1}{2\pi \sigma_{1}^{2}}{\sum_{i=1}^{m}\left\{y(x_{i},\omega )-t_{i}\right\}}^{2}-\frac{1}{2\pi \sigma_{2}^{2}}\omega^{T}\omega +c$$

where p(ω|t) is a posterior probability, p(t) is a constant independent of ω, *c* is a constant, and the prior probability corresponds to the L2 regular term in the ridge regression, and is thus called Bayesian ridge regression.

#### 2.2.4. AdaBoost Algorithm

The basic process of the AdaBoost algorithm is to train a group of component regressions in turn, in which the training set of each component regression is composed of the most informative samples given by the other component regressions. Finally, these component regressions are integrated with a linear weighting to obtain the final decision result [13]. The method for selecting the “richest information” sample is as follows. Each training sample is given a weight indicating the probability of it being selected for the training set by a component regression. For the regression prediction algorithms, first give a set of data sets $D=\{(x_{1},y_{1}),(x_{2},y_{2}),\ldots ,(x_{n},y_{n})\}$, the initialization sample weight is $C_{1}=\left\{C_{1i}=\frac{1}{n},i=1,2,\ldots n\right\}$, where *n* is the number of samples of the data set, and the weight set of the weak model is $P=\{P_{1},P_{2},\ldots ,P_{m}\}$, where *m* is the number of weak models constructed. The model error rate is calculated, assuming that the prediction class sequence of the training data set of the weak model M1 is P1, and the loss function of the prediction type sequence of the linear prediction data set is Pre_1.

(9) $$\max err=\{\max(|Y_{oi}-P_{1i}|),i=1,2,\ldots ,n)\}$$

(10) $$err_{i}=\frac{\left|Y_{oi}-p_{1i}\right|}{\max err}$$

The error rate formula, which is the weight of the weak model P1, is expressed as:

(11) $$err=\sum_{i=1}^{N}S1i\times erri,P_{1}=\ln\frac{1-err}{err}$$

The sample weights are updated using:

(12) $$C_{2i}=\frac{C_{1i}}{sum(C)}\times P_{1}^{1-erri},sum(C)=\sum_{i=1}^{n}S_{1i}\times P_{1}^{1-erri}.$$

Through successive iterations, different prediction results *pre_1*, *pre_2*, ..., *pre_m* and the weight set *P* of the model are obtained. When the maximum iteration number is satisfied and the iteration is stopped, the regression prediction result is expressed as:

(13) $$R_{i}=\sum_{k=1}^{m}P_{k}\times pre\_k_{i}$$

#### 2.2.5. AdaBoost Algorithm Optimized Using a Grid Search

A grid search is a kind of original digital programming method used to solve constrained nonlinear extremum problems. It also has no special requirements for functions [14]. Its optimization method involves dividing the problem into network lines within a certain range. The advantage of the grid search method is that it can search for several parameter values at the same time, eventually obtaining the optimal parameter combination for the evaluation function. In the process of optimization, the parameters of each group are decoupled from each other, which can avoid the problem of multiple solutions caused by many parameters or coupling between parameters, it is convenient for parallel calculation, and has a high efficiency.

**Figure 1:**
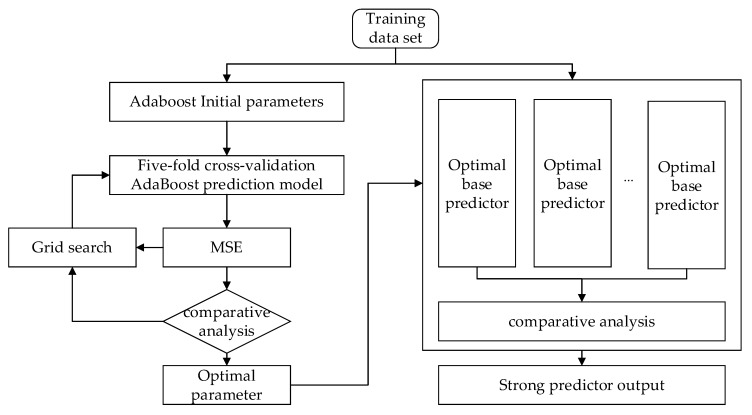
AdaBoost algorithm flowchart based on a grid search. MSE: Mean square error.

The prediction results of the AdaBoost algorithm are closely related to the base regression, the learning rate, the loss function, and the number of base regression loops; therefore, obtaining the optimal parameters is a critical step [15]. The grid search method first collects all possible parameter values and then groups them. Using the cross-validation grid search method, within the scope of the set of optimal model parameters, the mean square error (MSE) is compared for each forecast model such that it establishes the best prediction model that avoids the problem of the low accuracy of the original model. The flow of the AdaBoost algorithm based on grid search optimization is shown in Figure 1.

#### 2.2.6. Accuracy Evaluation

In this study, the three parameters selected to measure the accuracy of the evaluation models were the determination coefficient (*R*^2^), the root mean square error (*RMSE*), and the mean absolute error (*MAE*) [16]. The closer *R*^2^ is to 1, the higher the model fit and the more stable the model. The smaller the *RMSE* and *MAE* are, the higher the model prediction ability and the higher the model robustness. The larger *R*^2^ is, the smaller the *RMSE* and *MAE* are, and the higher the overall accuracy of the model [17].

(14) $$R^{2}=1-\frac{\sum_{i-1}^{n}{\left(\hat{y_{i}}-y_{i}\right)}^{2}}{\sum_{i-1}^{n}{\left(y_{i}-\bar{y}\right)}^{2}}$$

(15) $$RMSE=\sqrt{\frac{\sum_{i-1}^{n}{\left(y_{i}-\hat{y_{i}}\right)}^{2}}{n}}$$

(16) $$MAE=\frac{1}{m}\sum_{i=1}^{n}\left|y_{i}-\hat{y_{i}}\right|$$

Here, *n* is the number of samples, $y_{i}$ is the measured value, $\hat{y_{i}}$ is the predicted value, and $\bar{y}$ is the average of the measured values.

## 3. Results and Discussion

### 3.1. Soil Collection Preparation and Physical and Chemical Analysis

GPS positioning was adopted for the field collection of soil samples, where the coordinates of the actual sampling points and the detailed characteristic information of the sample plots were recorded. The surface soil with a depth of 0–20 cm was taken for each sample plot. To reduce the impact of rainfall and other factors, there was no precipitation in the first week of field sampling in the region and all sampling was completed within 1 days. According to the research needs, 41 samples were collected in the Honghu area and 28 samples were collected in northwest China [18]. After airdrying and sieving, the content of soil organic matter was determined using a potassium bichromate titrimetric method.

### 3.2. Spectral Reflectance Measurement

**Figure 2:**
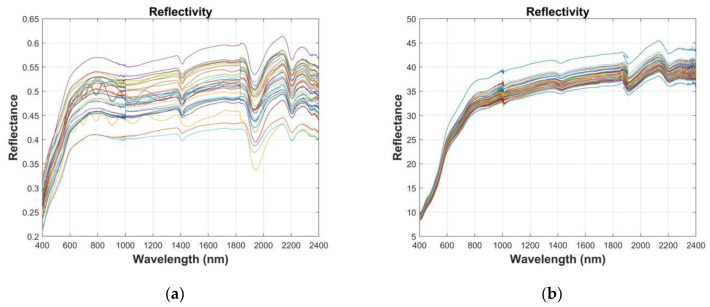
Soil reflectance spectra (with fringe noise removed): (**a**) northwest China and (**b**) Honghu.

The indoor spectral measurement was undertaken with an ASD FieldSpec 3 spectrometer (Analytical Spectral Devices Inc., Boulder, CO, USA). Its wavelength range is 350–2500 nm and its spectral resolution is 1 nm. The soil samples were placed in dark Petri dishes and the surface of the soil samples was scraped with a ruler. The light source was a halogen lamp with a power of 1000 W. The probe was positioned perpendicular to the soil surface, 10 cm above the surface layer [19]. Each soil sample was calibrated using a standard reference whiteboard before the spectral measurement to obtain a baseline [20]. To ensure the accuracy of the data, the reflectivity was measured in four directions (three rotations in the same direction, 90° each time), and each spectral curve was measured and averaged 10 times. The arithmetic mean of the four spectra was taken as the actual reflection spectrum data of the soil sample and the average reflectance of each soil sample was taken as the original reflectance spectrum value, which was done to reduce the interference and external noise. As can be seen in Figure 2, for each soil sample, we removed the noisy edge bands from 350–399 nm and 2400–2500 nm, and we retained the 400–2399 nm bands for the modeling analysis [21].

### 3.3. Calibration Set and Validation Set 

Before the modeling, the samples needed to be grouped. The 69 soil samples from the two research areas of northwest China and Honghu were divided according to a 3:1 ratio for the calibration set and validation set. The division of the calibration set and validation set was done using sample set partitioning based on the joint x-y distance (SPXY) method [22]. As can be seen from Table 1, 19 modeling sets and 9 validation sets were selected for the northwest China region, and 29 modeling sets and 12 validation sets were selected for the Honghu region.

### 3.4. Spectral Pretreatment

The spectral data were unstable due to the instrument itself and the data were inevitably affected by factors such as the test environment, the sample background, the observation angle, the smoothness of the sample surface, and stray light during the spectral acquisition process [23]. There are many methods used for spectral transformation processing. However, there is no transformation method suitable for each component. Therefore, it is necessary to try a variety of mathematical transformation methods to find the best treatment for a particular study [24].

**Figure 3:**
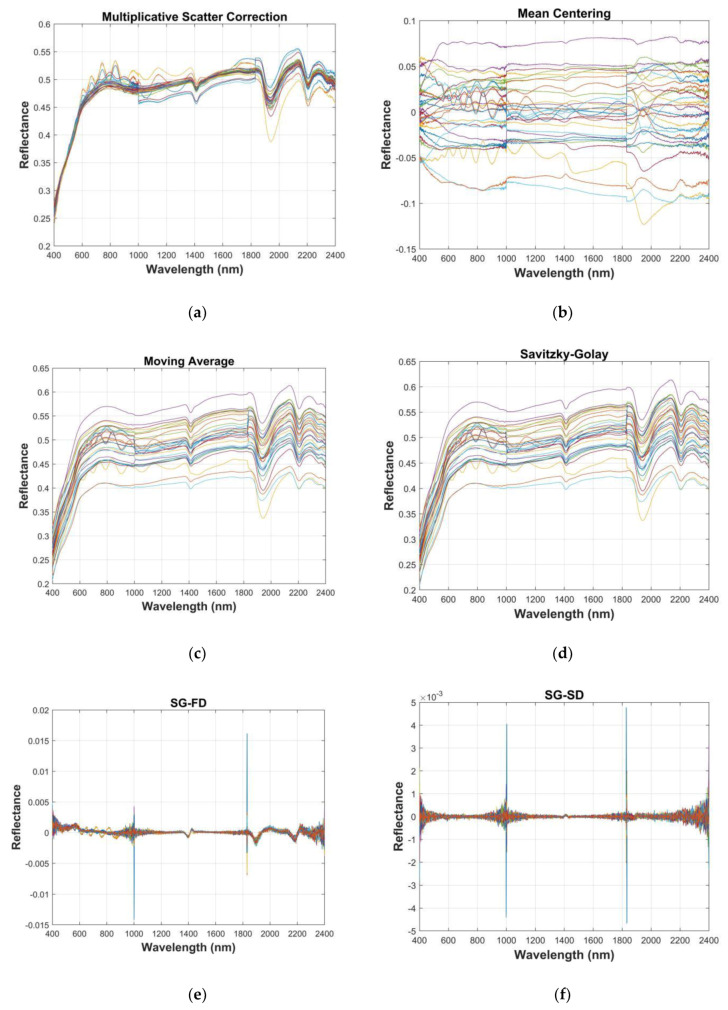

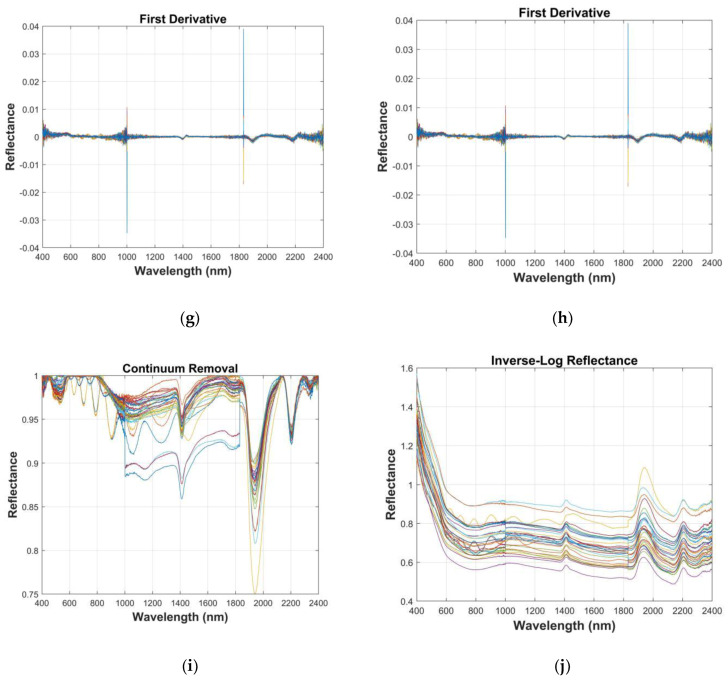
Soil pretreatment under different spectral indices (northwest China): (**a**) multiplicative scatter correction (MSC), (**b**) mean centering (MC), (**c**) moving average (MA), (**d**) Savitzky–Golay (SG), (**e**) first derivative after Savitzky–Golay (SG-FD), (**f**) second derivative after Savitzky–Golay (SG-SD), (**g**) first derivative (FD), (**h**) second derivative (SD), (**i**) continuum removal (CR), and (**j**) inverse-log reflectance (Log(1/R)).

**Figure 4:**
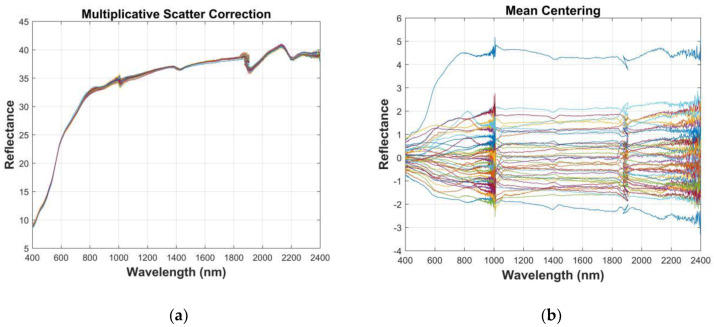

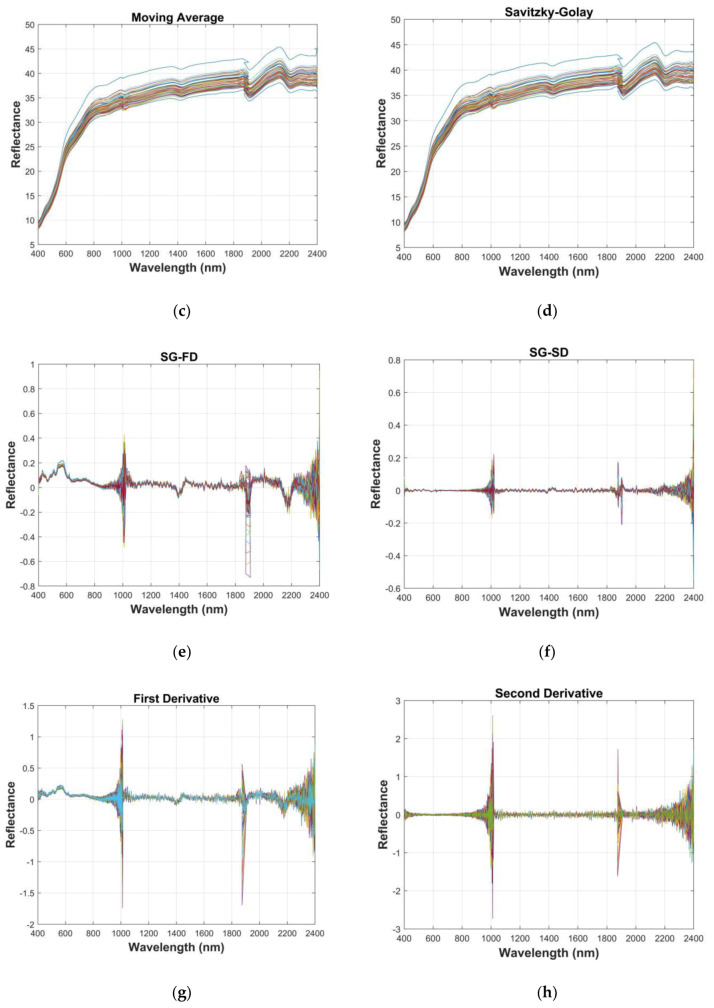

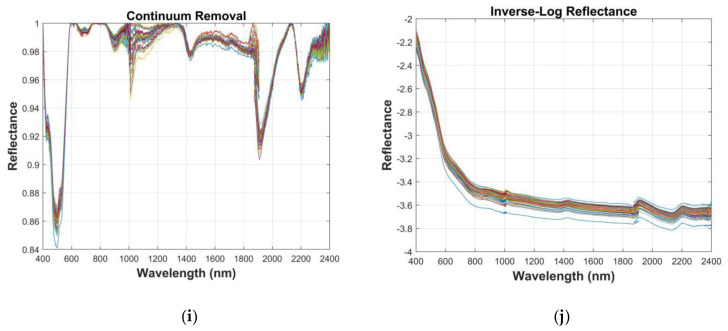
Soil pretreatment under different spectral indices (Honghu): (**a**) multiplicative scatter correction (MSC), (**b**) mean centering (MC), (**c**) moving average (MA), (**d**) Savitzky–Golay (SG), (**e**) first derivative after Savitzky–Golay (SG-FD), (**f**) second derivative after Savitzky–Golay (SG-SD), (**g**) first derivative (FD), (**h**) second derivative (SD), (**i**) continuum removal (CR), and (**j**) inverse-log reflectance (Log(1/R)).

It can be seen from Figure 3 (northwest China) and Figure 4 (Honghu) that the influence of parallel noise could be eliminated through differential processing of the spectral curve [24]. Ten spectral indicators were obtained: inverse-log reflectance (Log(1/R)), continuum removal (CR), multiplicative scatter correction (MSC), first derivative reflectance (FDR), second derivative reflectance (SDR), Savitzky–Golay (SG), first derivative after Savitzky–Golay (SG-FD), second derivative after Savitzky–Golay (SG-SD), moving average (MA), and mean centering (MC). The overlapped samples were separated to extract the spectral information, where there was little difference from the original data. Using the CR method to effectively restrain the background spectrum amplified the absorption spectrum information [25,26]. Generally speaking, by using 10 kinds of spectral preprocessing methods, the information of the spectral data was expanded to varying degrees.

### 3.5. Characteristic Band Selection

**Figure 5:**
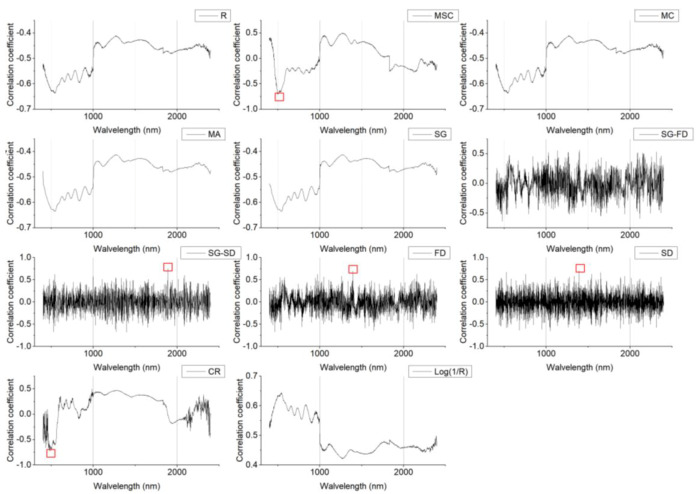
Changes in the soil organic matter (SOM) content and correlation coefficients represented using different spectral indices (Northwest China).

**Figure 6:**
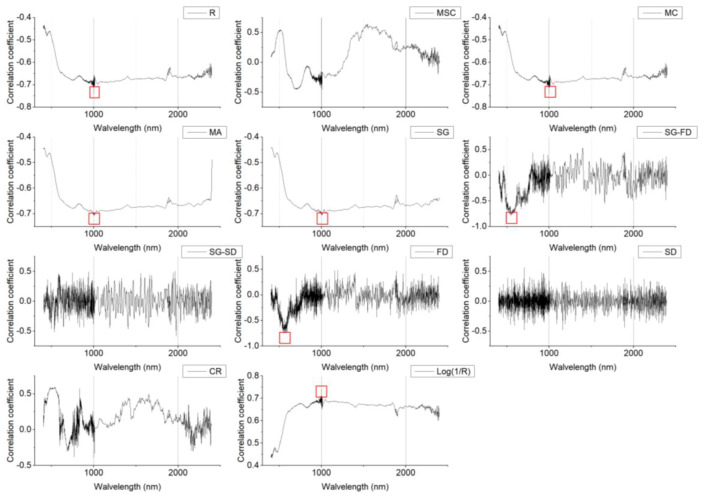
Changes in the SOM content and correlation coefficients represented using different spectral indices (Honghu area).

In this study, correlation analysis was carried out between the SOM content and the spectral transformation forms (MSC, MC, MA, SG, SG-FD, SG-SD, FD, SD, CR, Log(1/R)), as shown in Figure 5 and Figure 6. As can be seen in Figure 5, there was a certain degree of correlation between the SOM content and the smooth spectral reflectivity data, as well as the transformation form. The MSC spectra showed the largest negative correlations at 497 nm, 503 nm, and 504 nm, where the correlation coefficients were all greater than 0.7. Compared with the original spectrum, the SOM content was more closely correlated with the spectral data of the transformation forms. The correlation coefficient between the FD and SD spectra was the highest at 1393 nm and 1392 nm, where the correlation coefficients were 0.7 and 0.71. The SG-SD spectrum reached its highest positive correlation at 1888 nm, with a correlation coefficient of 0.76. The CR spectrum showed the largest negative correlation at 479 nm, 482 nm, and 487 nm, where the correlation coefficients were greater than 0.7. As can be seen in Figure 6, the spectral characteristic bands of the Honghu farmland showed different information. R, MC, MA, SG, and Log(1/R) showed good correlations in the range 974.1–1015.2 nm, the FD spectrum showed its highest negative correlation in the range 541.2–595.5 nm, and the SG-FD spectrum showed its highest negative correlation in the range 515.9–588 nm. 

According to the results of the single-band correlation analysis, as can be seen from Table 2, bands with an absolute correlation value greater than 0.7 were selected as the characteristic bands, where the corresponding mathematical transformation of the soil spectrum was used as the independent variable and the SOM content was used as the dependent variable to establish the hyperspectral quantitative prediction model of the SOM content.

### 3.6. Regression Model

To select the optimal regression model, RR, KRR, BRR, and AdaBoost regression were used in the comparison. Each regression model was set with the default parameters. The SOM content was used as the dependent variable and the measured spectrum was used as the independent variable to construct the regression model. The results are shown in Table 3, which show that: (1) by comparing eight regression models in two regions, it can be seen from the accuracy of the validation set that for northwest China, BRR achieved the best accuracy, while for the Honghu area, RR achieved the best accuracy; and (2) the Adaboost algorithm for unjoined grid search optimization performed the worst in different regions.

### 3.7. AdaBoost Algorithm Optimized Using a Grid Search

The grid search method was used in this study to find the best base regression, learning rate, loss function, and cycle number of the base regression. The search range and step size of each parameter in the grid search was initialized as follows: base estimators were RR, KRR, BRR, and a decision tree; value range of learning rate: 0.01–0.15, step size was set to 0.01; loss function: linear, square, and exponential; and value range of estimators: 50–300, step size was set to 50. The sample data was divided, the test error was calculated, five-fold cross-validation was selected, and after five training data iterations, the group parameter test results were selected as the mean values of the MSE. The optimal parameter combination was obtained from within the parameter range and the parameter combination was replaced. The average MSE value under all the parameter combinations in the grid was calculated successively. The parameter combination corresponding to the minimum MSE value was then obtained, which was the optimal parameter combination in the grid interval [27,28,29]. It can be seen from Table 4 that for the northwest China area, from the $R_{p}^{2}$ of the verification set, the maximum value of AdaBoost-KRR was 0.91, and the $RMSE_{p}$ and $MAE_{p}$ of the validation set had the same trend. For the Honghu area, from the $R_{p}^{2}$ of the verification set, the maximum value of AdaBoost-RR was 0.86, and the $RMSE_{p}$ and $MAE_{p}$ of the validation set had the same trend.

**Figure 7:**
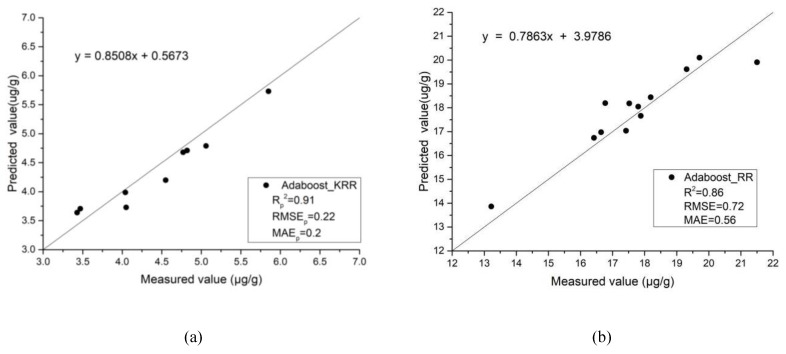
Comparison of the measured and predicted values of the different models:(**a**) northwest China and (**b**) Honghu.

Figure 7 compares the measured and predicted values of the different models. The x-axis shows the measured values of the SOM content and the y-axis shows the predicted values of the different models used for SOM content prediction. It can be seen that the overall accuracy of the models was high, and the relationship between the SOM content and measured hyperspectral reflectivity was well simulated.

## 4. Discussion

The correlation between an original reflectance and soil SOM content is not high. Different spectral transformations can enlarge and highlight the spectral characteristics of the original spectral reflectance and provide more characteristic bands on the original basis, which is conducive to the quantitative inversion of soil SOM content [24,30]. The correlation between spectral reflectance and soil SOM content can be effectively improved based on 10 spectral transformations, and the use of the higher correlation band can significantly improve the stability and prediction ability of the model [31].

In this study, an AdaBoost algorithm based on grid search optimization was introduced, and a variety of integrated learning soil organic matter quantitative inversion models were established. The prediction results of the AdaBoost algorithm based on grid search optimization and a single prediction model were compared and analyzed. From the results, the AdaBoost algorithm based on grid search optimization effectively improved the defect that a single prediction model has, namely that it easily falls into a local optimum. The results show that the algorithm effectively improved the prediction accuracy of the whole sample and the model generalization rate [29].

Compared with wide band remote sensing, hyperspectral remote sensing has the characteristics of high spectral resolution and strong band continuity. It can obtain more precise spectral information and is an important tool for the quantitative analysis of shallow soil properties. The spectral characteristics of soil spectral reflectance are caused by the mutual absorption and overlapping responses of different soil components. Estimations of specific soil attribute parameters can easily be affected by other soil components. Therefore, in this study, the combined spectral index model was used to focus on the mutual influence between bands, which can eliminate the mutual interference between band reflectance [32].

With the continuous progress of hyperspectral sensor technology, the difficulty of hyperspectral data acquisition is reduced. Therefore, the use of airborne or satellite hyperspectral sensors to obtain soil hyperspectral data has great potential for estimating regional soil surface organic matter content [33].

## 5. Conclusions

The general situations regarding the northwest China and Honghu study areas were as follows:

(1) The data quality had a significant impact on the modeling effect. By comparing the original spectral data and the correlation coefficient between the data transformed by the different spectral indices and SOM, it was found that the correlation was greatly improved after treatment, and the correlation between SOM and the measured spectrum increased. It is therefore important to study how to remove redundant data and improve the data quality.
(2) The regression ability of the ensemble learning algorithm was stronger than that of the traditional regression algorithm for the SOM spectrum. By using the AdaBoost algorithm based on a grid search, the prediction accuracy determination coefficient for the different regions could reach 0.85 or even higher. For the northwest China area, from the $R_{p}^{2}$ of the verification set, the maximum value of AdaBoost-KRR was 0.91, and for the Honghu area, from the $R_{p}^{2}$ of the verification set, the maximum value of AdaBoost-RR was 0.86, and the model had a better generalization ability.
(3) The experimental results prove that the hyperspectral technique is feasible for the analysis of the SOM content of soil. The different spectral index transformations combined with the Pearson correlation analysis spectral feature selection method solves the problem of information redundancy and poor prediction accuracy in the spectrum inversion domain. The proposed method could also be applied to the monitoring and identification of other agricultural soils.

## Figures and Tables

**Table 1:** Statistical characteristics of soil organic matter.

Study Area	Sample Type	Number	Minimum	Maximum	Mean	SD	CV (%)	Skewness	Kurtosis
Northwest China	Entire	28	3.43	7.34	4.6	0.8	17.4	1.47	4.1
Honghu	Entire	41	13.22	21.83	18.36	1.83	9.97	−0.46	0.4

**Table 2:** The feature bands and the correlation coefficients.

Study Area	Spectral Index	Characteristic Band Number	Characteristic Band Wavelengths (nm)	Maximum Correlation Coefficient	Minimum Correlation Coefficient
Northwest China	R	0		0.64	0.41
	MSC	3	497, 503, 504	0.70	0.00
	MC	0		0.64	0.41
	MA	0		0.64	0.41
	SG	0		0.64	0.41
	SG-FD	0		0.64	0.00
	SG-SD	1	1888	0.76	0.00
	FD	1	1393	0.70	0.00
	SD	1	1392	0.71	0.00
	CR	3	479, 482, 487	0.73	0.00
	Log(1/R)	0		0.64	0.42
Honghu	R	10	974.1–1009.2	0.74	0.43
	MSC	0		0.64	0.00
	MC	10	974.1–1009.2	0.74	0.43
	MA	13	994.8–1015.2	0.71	0.44
	SG	17	993.9–1011.2	0.71	0.44
	SG-FD	27	515.9–588	0.79	0.00
	SG-SD	0		0.58	0.00
	FD	7	541.2–595.5	0.76	0.00
	SD	0		0.57	0.00
	CR	0		0.60	0.00
	Log(1/R)	9	974.1–1006.5	0.74	0.43

**Table 3:** Results of the quantitative inversion models of SOM.

Study Area	Characteristic Bands	Models	$R_{p}^{2}$	$RMSE_{p}$	$MAE_{p}$
Northwest China	MSC_497_, MSC_503_, MSC_504_, SG-SD_1888_, FD_1393_, SD_1392_, CR_479_, CR_482_, CR_487_	RR	0.78	0.25	0.22
		KRR	0.87	0.20	0.16
		BRR	0.90	0.18	0.14
		AdaBoost	0.58	0.36	0.28
Honghu	R_974.1–1009.2_, MC_974.1–1009.2_, MA_994.8–1015.2_, SG_993.9–1011.2_, SG-FD_515.9–588_, FD_541.2–595.5_, Log(1/R)_974.1–1006.5_	RR	0.85	0.74	0.54
		KRR	0.77	0.93	0.68
		BRR	0.84	0.78	0.63
		AdaBoost	0.64	0.93	0.77

**Table 4:** The results of the quantitative inversion models of soil organic matter.

Study Area	Model	Learning Rate	Loss Function	Estimators	$R_{p}^{2}$	$RMSE_{p}$	$MAE_{p}$
Northwest China	AdaBoost-KRR	0.05	Square	300	0.91	0.22	0.20
Honghu	AdaBoost-RR	1.0	Linear	50	0.86	0.72	0.56
